# Additional Landing Sites for Recombination-Mediated Cassette Exchange in *C. elegans*

**DOI:** 10.17912/micropub.biology.000503

**Published:** 2021-12-16

**Authors:** Michael Nonet

**Affiliations:** 1 Dept of Neuroscience, Washington University Medical School, St. Louis MO 63110

## Abstract

Recombination-mediated cassette exchange (RMCE) is a recently developed alternative method for creating single copy transgenes using recombination rather than repair of double stranded breaks as the mechanism for driving integration into the genome. Two alternative methods for performing RMCE have been developed; a two-component approach using an unlinked source of FLP recombinase, and a one-component approach using a FLP expression cassette within the landing site. Here, I describe new landing sites for performing both types of RMCE. The new landing sites are located within 50 bp of well-vetted MosSCI insertion sites on Chr II and Chr IV.

**Figure 1. Overview of new RMCE landing sites:**
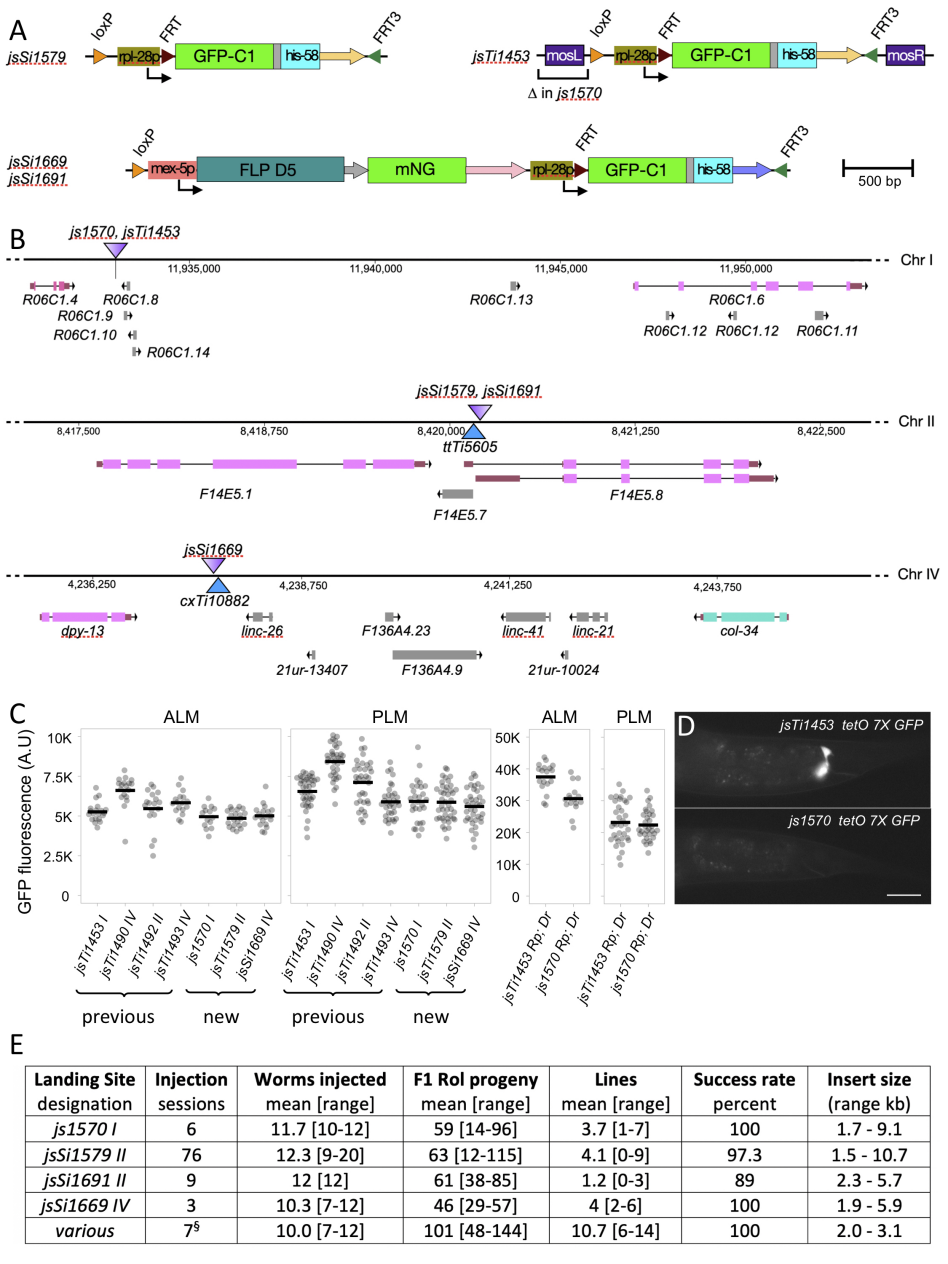
A) Structure of the *miniMos* and CRISPR/cas9 mediated single and two-component landing sites. Coding regions and *Mos1* transposase arms (thick rectangles), promoters (thin rectangles), and recombinase sites (triangles) are labelled. Unlabeled thick arrows represent 3′ UTRs: *unc-54* (yellow), *gpd-2/3* (grey), *his-58* (blue), *glh-2* (pink). The unlabeled grey region between *GFP-C1* and *his-58* is a flexible linker. *loxP*, *FRT,* and *FRT3* sites (triangles) not drawn to scale for clarity. B) Position and structure of the genomic interval of landing sites. *jsSi1579* is a two-component landing site integrated at a cas9 sgRNA site just adjacent to the position of the widely used *ttTi5605* *Mos1* insertion on Chr II. *jsSi1691* is a single-component landing site integrated at the same position as *jsSi1579*. *jsSi1669* is a single-component landing site integrated at a cas9 sgRNA site just adjacent to the position of the *cxTi10882* *Mos1* insertion on Chr IV. *js1570* is a derivative of *jsTi1453* on Chr I in which the *miniMos* left arm was deleted using CRISPR/cas9. *Mos1* insertions are represented by solid blue triangles. The landing site insertions are represented as triangles colored with a purple gradient oriented such that the dark side of the gradient represents the 5’ end of *GFP-his-58* coding sequences within the insertion and the light side represents the 3’ end. The position of the region on the chromosome (bp) is listed just below the line representing the chromosome. Coding genes are represented in pink and teal and non-coding genes in grey. Analogous schematics for the previously characterized landing sites are also available [See figure S2 of Nonet, (2020)]. C) Comparison of the expression levels of identical insertions at various RMCE landing sites. Expression level of a *mec-4p GFP-C1 tbb-2 3′* construct integrated at the previously described landing sites (Nonet, 2020) and the new Chr I, Chr II and Chr IV landing sites. I assumed that integration into *jsSi1579* and *jsSi1691* would yield the same expression level as they yield molecularly virtually identical insertions. The Chr II integration was made using *jsSi1579*. Removing the *mosL* arm from *jsTi1453* has only a minor effect on expression of an integrated *tetO 7X GFP* reporter (*Rp*) when driven by a *mec-4p* Tet OFF driver (*Dr*). Direct GFP fusion and bipartite reporter strains were imaged and quantified under the same conditions and thus can be directly compared. Strains imaged: NM5196, NM5209, NM5228, NM5236, NM5337, NM5467, NM5580, NM5582 and NM5633. n=14-21 for ALM and n=28-42 for PLM. D) Images of the gland cell background GFP expression of transgenic animals carrying *tetO 7X GFP-C1* integrated into *jsTi1453* andthe ∆mosL *js1570* derivative*.* Strains imaged: NM5264 and NM5327. Scale bar: *20 µm*. E) Frequency of insertions obtained at distinct landing sites. All injected animals were counted regardless of perceived quality of injection or survival. In most cases only a single gonad was injected. F1 Rol progeny were typically grouped 6 per plate for identification of integrated lines. Lines represents the number of independent F1 progeny plates that segregated an integrated line. Success is defined as obtaining an integration event at the expected genomic position. The insert size does not include the 7.85 kb vector and SEC sequences excised during heat shock. ^§^ Injections performed using PB washed DNA.

## Description

Transgenic animals are powerful tools in the study of basic biological processes using *C. elegans*. The recent development of recombination-mediated cassette exchange (RMCE) integration approaches in worms provides a relatively rapid efficient method to create single copy transgenes with inserts up to at least 12 kb (Nonet, 2020,Yang *et al.*, 2021). RMCE is similar to MosSCI (Frokjaer-Jensen *et al.*, 2008) in that it depends on integrating at landing sites that have been engineered. In the case of MosSCI, a transposon is excised, and the double stranded break is repaired by a template often using synthesis-dependent strand annealing (SDSA) which is error prone (Frokjaer-Jensen *et al.*, 2008). Similar approaches mediated by cas9 cleavage also have high error rates, approaching 65% in some studies (Au *et al.*, 2019). By contrast, RMCE uses recombination to insert the template into the genome which rarely yield erroneous inserts (Nonet, 2020).

The RMCE approach I developed takes advantage of two distinct recombinases. A plasmid template delivered into the gonad of young adult animals is first integrated into the genome using FLP recombinase. Both the template and the landing site contain two distinct FLP integration sites, *FRT* and *FRT3*. Recombination between the *FRT* and *FRT3* sites in the plasmid and the landing site yields replacement of the genomic *FRT FRT3* interval with plasmid *FRT FRT3* interval. This recombination likely occurs in two steps; first a loop in by recombination at one of the sites, followed by excision by recombination at the other. These recombination events typically occur in the F1 germline, though occasionally it occurs in the P0 animal (Nonet, 2020).

Two methods have been developed for performing RMCE. The first method uses a landing site and an unlinked source of FLP recombinase (usually *bqSi711*). Injection of the plasmid leads to integration of the plasmid at the landing site which is identified as a Rol (or Hyg^R^) animal. After the initial insertion is made homozygous, the self-excising marker cassette (SEC) is then excised using a heat shock Cre protocol, leading to the final insertion. The insertion is then outcrossed from *bqSi711* using simple crosses. The second method utilizes a landing site which contains a germline FLP expressing transcription unit contained within the landing site. In this case, the FLP expression element is excised using a heat shock Cre protocol. One limitation of the RMCE approach is the lack of landing sites for integration. Here, I describe several new landing sites created using a CRISPR integration approach.

I integrated a two-component landing site using CRISPR/cas9 just adjacent to the position of the *ttTi5605 Mos1* insertion that has been widely used for creating single copy insertions using MosSCI (Frokjaer-Jensen *et al.*, 2008). I also integrated a single-component landing site at same position on Chr II and another at a site adjacent to *cxTi10882*, another commonly used *Mos1* insertion site on Chr IV. In addition, I modified the previously described two-component landing site *jsTi1453 I*, deleting the left *miniMos*arm from that landing site (Fig. 1A, B). I first characterized these novel landing sites by integrating the identical *mec-4p GFP-C1 tbb-2 3’* construct at each site and comparing the expression level of insertions at the new landing sites to identical insertions at the previously described landing sites (Nonet, 2020). The expression of GFP-C1 in touch receptor neurons (TRN) was easily detected in all the new landing site transgenes, though the expression level was slightly lower than that observed in integrations at the four previously described sites (Fig. 1C).

Recently developed bipartite reporters including a *tetO*/tetR Tet OFF system exhibit background expression in both the pharynx and the rectal gland cells (Nonet, 2020). Because a similar rectal gland background signal was observed at several different landing sites using distinct bipartite systems, I speculated that the *miniMos* transposon arm might be contributing to the background. Comparison of the identical *tetO 7X ∆mec-7p GFP-C*1 reporter integrated at both *jsTi1453* and the *js1570 ∆mosL* arm derivative confirmed this was the case as expression in the rectal gland cells was undetectable in the *js1570* derived transgene (Fig 1D). Despite the reduction in background, the *tetO* reporter still robustly expressed GFP in TRNs, when driven by the identical *mec-4* promoter tet OFF driver (Fig. 1C).

In developing a new recombination-mediated homolog exchange technique (https://sites.wustl.edu/nonetlab/rmhe/), I have used these new landing sites to create addition RMCE insertions. I collated the insertion frequency data from a set of over 90 injection sessions in which I counted the number of Roller F1 animals obtained, and the number of insertions obtained (Fig. 1E). These data demonstrate that *js1570*, *jsSi1579* and *jsSi1669* all behave comparably to previously described landing sites, yielding insertions at a rate of approximately 1 per 3 injected animals. However, the *jsSi1691* single component site yielded insertions at a lower frequency (1 per 10 injected animals). I speculate this is due to lower expression of FLP from the *jsSi1691* landing site since integration at the same position using *jsSi1579* and *bqSi711* as a source of FLP yields normal integration frequencies. A recent study indicated that for CRISPR/cas9 genome modifications one could obtain a much higher frequency of integration events using specifically treated miniprep DNA (Huang *et al.*, 2021). A preliminary set of 7 injections performed while this manuscript was under review and presented in Figure 1E suggest that similar benefits are seen for RMCE, raising the insertion frequency to above 1 integration per P0 in this admittedly small sample size.

I previously demonstrated that RMCE yields insertions of expected structure in greater than 95% of cases (Nonet, 2020). I have also characterized most of the transgenes obtained at these additional landing sites either by confirming the presence of an expected fluorescence pattern, recombinase activity, *tetO* reporter activity, or *tetR* driver activity. In some cases, long range PCR combined with restriction digests and/or sequencing was also performed to confirm the structure of insertions. All of the insertions at *js1570* and *jsSi1669* have been verified. The analysis of insertions on Chr II is ongoing, but to date only one of over 50 well-characterized insertions is incorrect with that insertion containing a distinct region of Chr II inserted adjacent to the l*oxP* site. Three other cases of unexpected outcomes are also worth mentioning. In one case, I obtained a homozygous Rol insertion which still expressed the *rpl-28p* *GFP-his-58* marker from the landing cassette. In addition, in two cases, I was unable to excise the SEC by heat shock. However, in all three cases, an independent sister insertion from the same injection session was used for the excision step to isolate the final insertion. I have not attempted to determine the molecular structure of these unusual ‘faulty’ insertions since they are easily identified and discarded.

The new landing site are now available at the CGC and should provide additional flexibility in creating RMCE-based transgenic animals. I also plan to create additional landing sites at well-characterized high expressing genomic positions on the remaining chromosomes that currently do not contain landing sites.

## Methods

*C. elegans* was maintained on NGM agar plates spotted with OP50 at 22.5°C or at 25°C during the RMCE protocol.

RMCE transgenesis

Inserts were cloned into pLF3FShC (Nonet, 2020), pRMHEB or pRMHEP (https://sites.wustl.edu/nonetlab/rmhe-vectors/) and injected at ~50 ng/µl into young adults. In the set of injections summarized in Figure 1E Qiagen miniprep DNA was prepared using a PB wash as described by Huang *et al.* (2021) and injected at 40-50 ng/ul. Integrants were identified and isolated as described in detail in Nonet (2020). Performing RMCE at 25°C is critical to obtaining robust integration rates. The criterion for including an injection session in the table (Fig. 1E) was obtaining a mean of at least 1 F1 Rol per injected animal. All injection sessions into *js1570*, *jsSi1669* and *jsSi1691* met this criterion. Eleven *jsSi1579* injection sessions failed to meet this criterion. They consisted of 5 sessions injecting plasmids with strong ubiquitous promoters (*eft-3* or *rpl-27*) driving tet OFF and 6 sessions injecting plasmids that contained both a tet OFF driver and a *tetO* reporter cassette. In all cases dead eggs were observed on the injection plates. In cases where a fluorescent protein reporter was in the plasmid, the dead eggs were brightly fluorescent. The two *jsSi1579* failures included in the table were one *rpl-27* session and one dual driver and reporter plasmid session. In some cases, I was able to integrate plasmids containing both a driver and a reporter by injecting animals growing on doxycycline.

CRISPR/cas9-mediated insertions and deletion

*js1570* was created using a *dpy-10* co-CRISPR strategy. A mix of plasmids NMp3143 (40 ng/ul), NMp3153 (10 ng/ul), NMp3828 (20 ng/ul), NMp3829 (20 ng/ul) and oligonucleotides NMo5238 (0.4 uM) and NMo6761 (0.4 uM) we co-injected into *jsTi1453; bqSi711* animals. Rol progeny were screen by PCR (NMo6564/6569) for presence of the deletion and homozygosed. The deletion structure was confirmed by sequence analysis. *jsSi1579* was created by injecting *unc-119(ed3); bqSi711* animals with a mix of NMp3143 (40ng/ul), NMp3630 (50 ng/ul), NM3631 (30 ng/ul). Insertions were identified by selection for hyg^R^ Rol progeny by adding 25 ul of 100 mg/ml HydroGold™(InvivoGen) to P0 injection plates (3 animals per plate) 3 days after injection. After isolating homozygotes, the *hyg^R^ sqt-1* self-excision cassette (SEC) in the insertion was excised by screening for non-Rol progeny after a 20-hr. heat shock at 29° C. The structure of the insertion was validated by a combination of long-range PCR [performed as outlined in Nonet (2020) using MNo3887/3888], restriction digestion and sequence analysis. *jsSi1691* was created by injection of N2 (the wild type) with a mixture of plasmids NMp3143 (40 ng/ul), NMp3630 (50 ng/ul), NMp4043 (20 ng/ul), pBluescript KS (50 ng/ul), pGH8 (2 ng/ul) and pCFJ90 (2.5 ng/ul). Insertions were selected for and analyzed as described for *jsSi1579*. *jsSi1669* was created by injection of *oxTi1127* animals with a mixture of plasmid NM4055 (35 ng/ul), NM4057 (25 ng/ul), pBluescript KS (+) (50 ng/ul), pGH8 (2 ng/ul) and pCFJ90 (2.5 ng/ul). Insertions were identified by hygromycin selection as described above. The structure of the insertion was validated as described above using oligonucleotides NMo3889/3890. The genomic sequence of the insertions is available at https://sites.wustl.edu/nonetlab/rmce-insertion-strains/ .

Microscopy

For quantification of GFP signals, homozygous L4 hermaphrodite animals were mounted on 2% agar pads in a 2 µl drop of 1mM levamisole in phosphate buffered saline, cover slipped and imaged on an Olympus (Center Valley, PA) BX-60 microscope equipped with a Qimaging (Surrey, BC Canada) Retiga EXi monochrome CCD camera, a Lumencor AURA LED light source, Semrock (Rochester, NY) GFP-3035B and mCherry-A-000 filter sets, and a Tofra (Palo Alto, CA) focus drive, run using micro-manager 2.0ß software (Schindelin *et al.*, 2012) using a 40X air lens at 20% LED power with 100 ms exposures. PLM soma and ALM soma signals were quantified using the FIJI version of ImageJ software (Edelstein *et al.*, 2014) as described in Nonet (2020). Rectal gland cell images presented were collected using the same conditions.

Plasmid constructions

Integration vectors were assembled using Golden Gate (GG) reactions as described in Nonet (2020). Other plasmids were constructed using standard cloning techniques.

The following previously published plasmids were used:

pBluescript KS (+) (Short *et al.*, 1988), pDD162 (Dickinson *et al.*, 2013), pCFJ90 and pGH8 (Frokjaer-Jensen *et al.*, 2008), and NMp3055, NMp3421, NMp3467, NMp3470, NMp3631, NMp3643, NMp3732, NMp3746 and NMp3774 (Nonet, 2020).

The following plasmids were constructed:

NMp3143 peft-3-cas9 (3 int)

A derivative of the pDD162 Cas9 expression plasmid lacking the empty U6 sgRNA cassette. pDD162 was amplified using NMo5228/5379 and re-circularized using a Gibson assembly reaction.

NMp3150 DR274 U6 FE

U6 promoter sgRNA clone with a flipped and extend sgRNA as described in Ward (2015). sgFE was amplified from NMp3055 using NMo5407/5075, purified, digested with BamHI and HindIII, and inserted into BamHI and HindIII digested NMp3055.

NMp3153 DR274 U6 FE dpy-10

U6 promoter sgRNA targeting *dpy-10* at GCTACCATAGGCACCACgAG. NMp3150 was digested with BsaI and the annealed oligonucleotide pair NMo5236/5237 was inserted by ligation.

NMp3630 DR274 U6 MosII

U6 promoter sgRNA targeting Chr II adjacent to the *ttTi5605* *Mos1* insertion site at gatatcagtctgtttcgtaa cgg. NMp3055 was digested with BsaI and the annealed oligonucleotide pair NMo6439/6440 was inserted by ligation.

NMp3828 DR274 U6 sgTi1453 F

U6 promoter sgRNA targeting Chr I adjacent to the *jsTi1453* landing site at attcacggcacaacatacat tgg. NMp3055 was digested with BsaI and the annealed oligonucleotide pair NMo6757/6758 was inserted by ligation.

NMp3829 DR274 U6 sgMosL

U6 promoter sgRNA targeting *miniMos* adjacent to the left arm at gttgAGCTCCACCGCGGTGG CGG. NMp3055 was digested with BsaI and the annealed oligonucleotide pair NMo6759/6760 was inserted by ligation.

NMp4043 pSAP ChrII FLP loxP FRT FRT3 landing

Chr II full RMCE landing site CRISPR template. The left arm amplified from N2 genomic DNA using NMo6707/6450, the right arm amplified from N2 genomic DNA using NM06708/6453, and the loxP FLP FRT FRT landing site from NMp3746 were co assembled into NMp3421 using a SapI Golden Gate assembly reaction.

NMp4053 DR274 5′ arm cxTi10882 left arm

Left arm genomic fragment adjacent to *cxTi10882*. The left arm was amplified from N2 genomic DNA using NMo7060/7061 and inserted into NMp3467 using a BsaI Golden Gate reaction.

NMp4054 DR274 3′ arm cxTi10882 right arm

Right arm genomic fragment adjacent to *cxTi10882*. The right arm was amplified from N2 genomic DNA using NMo7058/7059 and inserted into NMp3470 using a BsaI Golden Gate reaction.

NMp4055 DR274 U6 cxTi10882

U6 promoter sgRNA targeting Chr IV adjacent to the *cxTi10882* Mos1 insertion site at actgttggatgcctgtgtag cgg. NMp3055 was digested with BsaI and the annealed oligonucleotide pair NMo7062/7063 was inserted by ligation.

NMp4057 pSAP cxTi10882 FLP loxP FRT FRT3 landing

Chr IV full RMCE landing site CRISPR template. The left chr IV arm from NMp4053, the right Chr IV arm from NMp4054 and the loxP FLP FRT FRT landing site from NMp3746 were co assembled into NMp3421 using a SapI Golden Gate assembly reaction.

Oligonucleotides

**Table:** 

**NMo number**	**Sequence 5’ > 3’**
3887	ACCGGAAACCAAAGGACGAGAG
3888	ACGCCCAGGAGAACACGTTAG
3889	CCAAACAAGTGTCGTTGACCCAG
3890	CATATCCGCCAAGGACGCTC
5075	GCCAAGCTTCACAGCCGACTATGTTTGGCGTC
5228	CGCCAGGGTTTTCCCAGTCACGACGTTGTAAAACGACGGCCAGTG
5236	TTTGCTACCATAGGCACCACGAG
5237	AAACCTCGTGGTGCCTATGGTAG
5238	ACTTGAACTTCAATACGGCAAGATGAGAATGACTGGAAACCGTACCGCATGCGGTGCCTATGGTAGCGGAGCTTCACATGGCTTCAGACCAACAGCCTAT
5379	CGTCGTGACTGGGAAAACCCTGGCGTTCCCAACAGTTGCGCAGCC
5407	AAGGATCCGGGTCTCAGTTTAAGAGCTATGCTGGAAACAGCATAGCAAGTTTAAATAAGGCTAGTCCG
6439	TTTGATATCAGTCTGTTTCGTAA
6440	AAACTTACGAAACAGACTGATAT
6450	TTGCTCTTCATGGCCTCTGAACTGGTACCTC
6453	TTGCTCTTCATACCTTGCCATTGTTTCCTG
6564	CATCCCATTCACGGCACAAC
6569	AAGCTCTTCACTCCGCATTTTCTCCCACCCTG
6707	AAGCTCTTCACGCGAAACAGACTGATATCGAAAC
6708	AAGCTCTTCAACGTAACGGTCTTCTGTATAAC
6757	TTTGATTCACGGCACAACATACAT
6758	AAACATGTATGTTGTGCCGTGAAT
6759	TTTGATTCACGGCACAACATACAT
6760	AAACATGTATGTTGTGCCGTGAAT
6761	AACAATTCATCCCATTCACGGCACAACATATGGCGGCCGCTCTAGAACTAGGCTGTTTCG
7058	AGGTCTCAGACGCTGTGTAGCGGTCCTCTATTG
7059	AGGTCTCTCTACAGTCGCATACGTCGTATCCC
7060	GGTCTCAGTGGTTTCAACGGTGGAAGAAGGG
7061	CGGTCTCTTCGCACAGGCATCCAACAGTACG
7062	TTTGACTGTTGGATGCCTGTGTAG
7063	AAACCTACACAGGCATCCAACAGT
	

Novel transgenes

**Table:** 

**Transgene**	**Description**	**Full Designation**	**Comments**
*js1570 I*	Chr I landing site	*jsTi1453 js1570 [∆mosL loxP rpl-28p FRT GFP-his-58 FRT3 mosR] I*	This study
*jsSi1579 II*	ChrII landing site	*jsSi1579 [loxP rpl-28p FRT GFP-his-58 FRT3] II*	This study
*jsSi1669 IV*	Chr IV landing site	*jsTi1669 [loxP mex-5p FLP D5 sl2 mNG glh-2 3′ rpl-28p FRT GFP-his-58 FRT3] IV*	This study
*jsSi1691 II*	Chr II landing site	*jsTi1691 [loxP mex-5p FLP D5 sl2 mNG glh-2 3′ rpl-28p FRT GFP-his-58 FRT3] II*	This study
*jsSi1571 I*	7X tetO GFP-C1	*jsTi1453 js1570 jsSi1571 [∆mosL loxP tetO 7X ∆mec-7p GFP-C1 tbb-2 3′ FRT3 mosR] I*	This study. RMCE insertion of NMp3774 into *js1570*
*jsSi1684 IV*	*mec-4p* GFP-C1	*jsTi1669 jsSi1684 [loxP mec-4p GFP-C1 tbb-2 3′ FRT3] IV*	This study. RMCE insertion of NMp3732 into *jsSi1669*
*jsSi1707 II*	*mec-4p* GFP-C1	*jsSi1579 jsSi1707 [loxP mec-4p GFP-C1 tbb-2 3′ FRT3] II*	This study. RMCE insertion of NMp3732 into *jsSi1579*
*jsSi1734 I*	*mec-4p* GFP-C1	*jsTi1453 js1570 jsSi1734 [∆mosL loxP mec-4p GFP-C1 tbb-2 3′ FRT3 mosR] I*	This study. RMCE insertion of NMp3732 into *js1570*

Worm Strains

**Table:** 

**Strain**	**Genotype**	**Source**
BN711	*unc-119(ed3) III; bqSi711 [mex-5p::FLP::SL2::mNG + unc-119(+)] IV*	Macías-León and Askjaer (2018); CGC
EG8992	*F53A2.9(oxTi1127[Pmex-5::Cas9::tbb-2 3’UTR, Phsp-16.41::Cre::tbb-2 3’UTR, Pmyo-2::nls-CyOFP::let-858 3’UTR + lox2272]) III*	Schwartz *et al.* (2021); CGC
NM5196	*jsTi1493 jsSi1502 [mosL loxP mec-4p GFP-C1 tbb-2 3′ FRT3 mosR] IV*	Nonet (2020); CGC
NM5209	*jsTi1453 jsSi1514 [mosL loxP mec-4p GFP-C1 tbb-2 3′ FRT3 mosR] I; him-8(e1489) IV*	Nonet (2020)
NM5228	*jsTi1490 jsSi1529 [mosL loxP mec-4p GFP-C1 tbb-2 3′ FRT3 mosR] IV*	Nonet (2020); CGC
NM5236	*jsTi1492 jsSi1535 [mosL loxP mec-4p GFP-C1 tbb-2 3′ FRT3 mosR] II*	Nonet (2020)
NM5264	*jsTi1453 jsSi1543 [mosL loxP tetO 7X ∆mec-7p GFP-C1 tbb-2 3′ FRT3 mosR] I; him-8(e1489) IV*	Nonet (2020)
NM5295	*jsTi1493 jsSi1560 [mosL loxP mec-4p tetR-L-QF_AD_act-4 3′ FRT3 mosR] IV*	Nonet (2020)
NM5322	*jsTi1453 js1570 [∆mosL loxP rpl-28p FRT GFP-his-58 FRT3 mosR] I; bqSi711 IV*	This study
NM5327	*jsSi1453 js1570 jsSi1571[loxP tetO 7X ∆mec-7p GFP-C1 tbb-2 3′ FRT3 mosR] I; him-8(e1489) IV*	This study
NM5337	*jsSi1453 js1570 jsSi1571 [loxP tetO 7X ∆mec-7p GFP-C1 tbb-2 3′ FRT3 mosR] I; jsTi1493 jsSi1560 [mosL loxP mec-4p tetR-L-QF_AD_ act-4 3′ FRT3 mosR] IV*	This study
NM5402	*jsSi1579 [loxP rpl-28p FRT GFP-his-58 FRT3] II; bqSi711 IV*	This study
NM5467	*jsTi1453 jsSi1543 [mosL loxP tetO 7X ∆mec-7p GFP-C1 tbb-2 3′ FRT3 mosR] I; jsTi1493 jsSi1560 [mosL loxP mec-4p tetR-L-QF_AD_ act-4 3’ FRT3 mosR] IV*	Nonet (2021)
NM5471	*jsTi1669 [loxP mex-5p FLP D5 sl2 mNG glh-2 3′ rpl-28p FRT GFP-his-58 FRT3] IV*	This study
NM5500	*jsTi1691 [loxP mex-5p FLP D5 sl2 mNG glh-2 3′ rpl-28p FRT GFP-his-58 FRT3] II*	This study
NM5580	*jsTi1579 jsSi1707 [loxP mec-4p GFP-C1 tbb-2 3′ FRT3] II*	This study
NM5582	*jsTi1669 jsSi1684 [loxP mec-4p GFP-C1 tbb-2 3′ FRT3] IV*	This study
NM5633	*jsTi1453 js1570 jsSi1734 [∆mosL loxP mec-4p GFP-C1 tbb-2 3′ FRT3 mosR] I; him-8(e1489) IV*	This study

## Reagents

Plasmids are available by request from MLN. Strains containing the four new landing sites have been submitted to the *Caenorhabditis* Genetics Center (CGC). Plasmids and additional strains and will be submitted to Addgene or CGC and if demand levels warrant it.
